# Random Forest Modelling of Milk Yield of Dairy Cows under Heat Stress Conditions

**DOI:** 10.3390/ani11051305

**Published:** 2021-04-30

**Authors:** Marco Bovo, Miki Agrusti, Stefano Benni, Daniele Torreggiani, Patrizia Tassinari

**Affiliations:** Department of Agricultural and Food Sciences—Agricultural Engineering (DISTAL), Alma Mater Studiorum University of Bologna, Viale Fanin 48, 40127 Bologna, Italy; miki.agrusti@unibo.it (M.A.); stefano.benni@unibo.it (S.B.); daniele.torreggiani@unibo.it (D.T.); patrizia.tassinari@unibo.it (P.T.)

**Keywords:** livestock sustainability, precision livestock farming, heat stress, random forest, machine learning

## Abstract

**Simple Summary:**

Sustainability is a necessary goal for animal-derived products due to the mounting pressure on the livestock sector to meet the growing demand of an increasing population with rising incomes and the need to reduce the exploitation of resources and environmental impact, while safeguarding animal welfare. We found that by considering a precision livestock farming approach to feeding, advanced numerical methods could represent a reliable and viable tool for the evaluation of future productive scenarios of dairy cows in the presence of changing climate conditions. We believe that the model proposed here could help to develop and improve decision support for farmers to increase both milk yield and animal welfare and, on the other hand, to reduce the resources needed, hence increasing sustainability of the dairy sector.

**Abstract:**

Precision Livestock Farming (PLF) relies on several technological approaches to acquire, in the most efficient way, precise and real-time data concerning production and welfare of individual animals. In this regard, in the dairy sector, PLF devices are being increasingly adopted, automatic milking systems (AMSs) are becoming increasingly widespread, and monitoring systems for animals and environmental conditions are becoming common tools in herd management. As a consequence, a great amount of daily recorded data concerning individual animals are available for the farmers and they could be used effectively for the calibration of numerical models to be used for the prediction of future animal production trends. On the other hand, the machine learning approaches in PLF are nowadays considered an extremely promising solution in the research field of livestock farms and the application of these techniques in the dairy cattle farming would increase sustainability and efficiency of the sector. The study aims to define, train, and test a model developed through machine learning techniques, adopting a Random Forest algorithm, having the main goal to assess the trend in daily milk yield of a single cow in relation to environmental conditions. The model has been calibrated and tested on the data collected on 91 lactating cows of a dairy farm, located in northern Italy, and equipped with an AMS and thermo-hygrometric sensors during the years 2016–2017. In the statistical model, having seven predictor features, the daily milk yield is evaluated as a function of the position of the day in the lactation curve and the indoor barn conditions expressed in terms of daily average of the temperature-humidity index (THI) in the same day and its value in each of the five previous days. In this way, extreme hot conditions inducing heat stress effects can be considered in the yield predictions by the model. The average relative prediction error of the milk yield of each cow is about 18% of daily production, and only 2% of the total milk production.

## 1. Introduction

Sustainability is an unavoidable goal for animal-derived products due to the mounting pressure on the livestock sector to meet the growing demand of an increasing population with rising incomes and the need to reduce the exploitation of resources and the environmental impact, while safeguarding animal welfare [1]. At the same time, global climate change and environmental crises are also challenging the dairy sector, and they will represent increasingly important issues to be addressed to ensure its economic, environmental, and social sustainability. In the dairy sector, the cornerstones of sustainability can be recognized as milk production and quality, cow health and welfare, efficiency in the use of resources, and emissions reduction. Animal welfare is strictly related to sustainability, due to the consequences in terms of milk quantity and quality, which affect the efficiency of the use of natural resources. For this purpose, a crucial point is the prevention of heat stress, as it markedly jeopardizes animal welfare in several countries in the Mediterranean area.

At the same time, equipment based on Information and Communication technology (ICT) are increasingly installed in livestock barns to perform a wide range of operations—from climatic control to milking, from precision feeding to cleaning [2]. These devices are coupled with manifold sensors which collect data needed for a proper operation of the equipment and, therefore, large amounts of data are recorded nowadays in a livestock farm equipped with ICT systems. This is a significant aspect of the Precision Livestock Farming (PLF) approach, which is involving the livestock farming sector in a fast process and is providing farms with great opportunities of improvement of the production performance and the conditions of animal welfare, independently of the farm size [3,4]. In the dairy cattle sector, the availability of data recorded in real time concerning the environmental conditions of the barn and the production performances of the individual cows represent a quantitative knowledge basis with a huge potential of development of further informatic and electronic tools, able to achieve optimal conditions of animal welfare and more sustainable productions, in addition to improvements in milk quality and production efficiency [5]. In particular, the ever more widespread Automatic Milking Systems (AMSs) provide farmers with detailed data concerning health conditions and parameters connected to the milk produced, which are of great interest to optimize the production [6,7]. Moreover, in technological farms, data concerning different parameters of behavior and activity of cows, animal health and welfare are collected from different sensors (e.g., individual cow data recording system, activity tags such as pedometers or neck collars, ear tags for rumination monitoring, automatic concentrate feeders), and used for the daily management of the herd [8].

To gain a comprehensive understanding of these phenomena and monitor and control the production processes in relation to climate change in a sustainable intensification perspective, sophisticated and high-throughput data acquisition is needed, providing very heterogeneous and multichannel datasets. Several studies have shown that a proper storage of collected data in structured databases represents a necessary preliminary step for the development of numerical models suitable to characterize the conditions and performance of individual cows [9] and to quantify the effects of particular thermo-hygrometric conditions on milk production [10,11]. In a climate change scenario, the welfare of dairy cows exposed to heat waves is becoming increasingly important [12]. Moreover, cow activity response to heat load was recently investigated [13]. Cows in the advanced lactation stage proved to be more sensitive to heat load than cows in early lactation. Moreover, multiparous cows showed less pronounced activity responses than primiparous ones. In fact, heat load accumulation and individual cow-related factors proved to be significant factors for prediction models based on the individual susceptibility of animals to heat stress [14,15]. Applied statistical methods used in the literature [16] showed that milking frequency, lactation number (parity number), month of milking, and type of lying stall represent important factors responsible for the monthly milk yield of dairy cows in farms with AMSs. In this context, Machine Learning (ML) algorithms have been already applied in some areas of dairy research, particularly to predict data, and they represent a promising tool, useful to develop and improve decision support for farmers [17] in order to increase both milk yield and animal welfare and, on the other hand, to reduce the resources needed, hence increasing the sustainability of the sector [18,19].

Further studies are thus necessary to identify how factors related to animal welfare and cow performance can be combined with indoor conditions inside the barn. To this end, continuous and real-time monitoring of the animals and the environmental parameters of the barn contributes to the knowledge of the welfare conditions of the individual cows: it can provide important information for the management of the barn environment [20] and for the prevention of problems related to the longevity of the cows, their productivity, and the quality of the milk.

The study aims to define, train, and test a model developed through machine learning techniques and, in particular, by adopting a Random Forest algorithm, having the main goal to assess the trend in daily milk yield of a single cow in relation to environmental conditions. The model can be applied as a regression tool or as predictive tool. In the paper, we will show that the methodology proposed here can be applied for both purposes by considering three different numerical scenarios. In the statistical model, having seven predictor features, the daily milk yield is evaluated as a function of the position of the day in the lactation curve and the indoor barn conditions expressed in terms of daily average of the temperature-humidity index (THI) in the same day and its value in each of the five previous days, recognized as a statistically significant period for the production on the day under consideration. In this way, extreme hot conditions inducing heat stress effects can be considered in the yield predictions by the model. The model has been calibrated and tested on the data collected on 91 lactating cows of a dairy farm, located in northern Italy and equipped with an AMS and two thermo-hygrometric sensors acquiring information on the environmental conditions, during two entire years—i.e., 2016 and 2017. To validate and test the forecasting potentials of the method, as well as to quantify its reliability, three different numerical scenarios, i.e., A, B, and C, have been considered. Scenario A has the objective to test the model for regression purposes, while B and C aim to evaluate the reliability of the model in providing the time series trend of future milk yields.

## 2. Materials and Methods

### 2.1. Housing and Animals

The data collection and the validation of the model have been carried out with reference to a case study dairy farm located in the municipality of Budrio, about 15 km NE of Bologna (Italy). The region is characterized by hot summer seasons with high percentage of humidity; in fact, considering the warmer months of the year (i.e., June, July and August), the average of the daily maximum temperature typically ranges from 27 to 29 °C, with daily average relative humidity, for the same period, from 75% to 85%.

**Figure 1:**
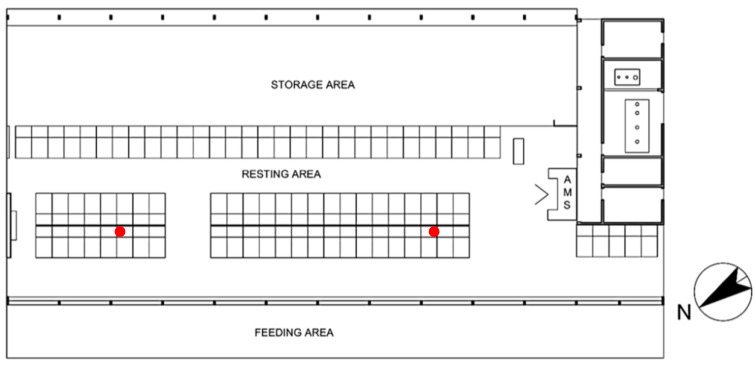
Layout of the case study barn (AMS: Automatic Milking System; ● position of the two thermo-hygrometer data loggers).

The rectangular layout of the barn is 51 m long and 23 m wide, with the longitudinal axis SW–NE-oriented, a ridge height of 8.52 m and gutter heights of 4.95 m on the NW side and 6.65 on the SE side. It consists of a hay storage area on the SE side, a resting area in the central zone of the building and a feeding area with feed delivery lane on the NW side (see Figure 1). The resting area has a partially slatted floor and hosts 78 cubicles with straw bedding. Two blocks of head-to-head rows are in the central part of the resting area, while another row runs along the entire length of the barn close to the storage area. Mechanical ventilation is controlled by three high volume low speed (HVLS) fans with five horizontal blades which were activated by a temperature-humidity (TH) sensor situated in the middle of the barn at about 3 m of level.

Lactating cows are fed with a total mixed ratio kept available along the feeding lane. About 65 Friesian cows are milked everyday using an AMS “Astronaut A3 Next” (Lely, Maassluis, The Netherlands) placed at the SW extremity of the barn. During the period of the study, the robot was programmed to ensure a particular number of daily visits for each cow depending on her productivity and her expected optimal milk yield per visit, with a minimum of two and a maximum of four daily visits as constraints. Animals with fewer than two visits in one day were signaled by a warning, while the cows which have been milked four times in one day can only pass through the AMS box without being milked and fed further. The milk room is located on the SW side of the building, next to the offices and the technical rooms. The robot also manages the supplement feeding, which is calculated based on daily milk yield and days in milk (DIM) value: it linearly increases with time from 3.0 kg to 3.5 kg for cow during the first 15 DIM, then it is proportional to milk yield with a coefficient 0.157 kg/L up to the limit of 7.50 kg. Finally, during the last 14 days of the milking period, the supplementation decreases linearly with time to the lower limit of 1.5 kg.

### 2.2. Milk Yield and Environmental Data

The period of study spans the years of 2016 and 2017. In this period, 132 cows were milked by the AMS system, although 91 animals were considered in the study—i.e., the cows with more than 100 daily milk production values. All of this ensured a robust dataset for each cow necessary to perform a reliable training of the numerical model described in the following. Among the 91 cows, at the beginning of the study, 41 were single-parity and 50 were multiparity. The data of the various milking events recorded by the AMS were downloaded, together with the cow tags and the DIM in a large dataset. Then, the daily milk yields were calculated for each cow. The dataset was then filtered by eliminating the exceptional events (e.g., daily milk yields of cows with mastitis or other factors that can influence animal production). This allowed us to create a cleaned dataset for each cow, collecting the time series of the milk yields during the monitored period. The cow datasets considered in the study range from 100 to about 550 milk daily yields.

As far as the recording of environmental data is concerned, two thermo-hygrometer data loggers, PCE-HT71, with an accuracy of ±3% on the relative humidity (*RH*) and ±1 °C on the temperature *T*, were positioned inside the barn (see Figure 1) and recorded the indoor temperature *T_in_* and relative humidity *RH_in_* from 1 January 2016 to 31 December 2017. Outdoor thermo-hygrometric parameters were measured, for the same period, by a weather station located in the proximity of the building. The thermo-hygrometric data loggers recorded temperature and humidity at 30 min intervals and for each couple of values the THI was calculated following Equation (1), described by the National Research Council [21]:

THI = [(1.8 × *T_db_* + 32) − (0.55 − (0.0055 × *RH*) × (1.8 × *T_db_* − 26)] (1)

where *T_db_* is the dry bulb temperature (*T_db_* in °C) and RH is the relative humidity (RH in %).

**Figure 2:**
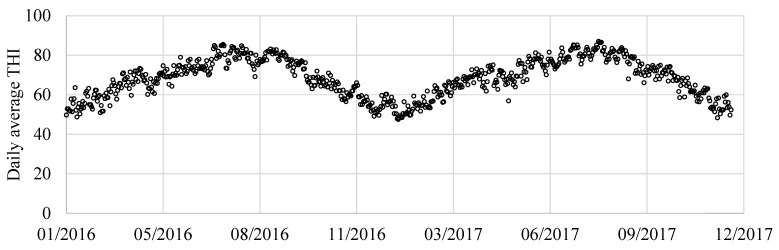
Barn indoor THI values calculated for the years 2016–2017 considered in the study.

Then, the daily average THI was calculated for the two thermo-hygrometer sensors. The values of the two showed a maximum difference of only 0.8, confirming the environmental homogeneity in the barn. In the study, the mean values of THI obtained by the two thermo-hygrometers were considered. They are showed in Figure 2.

### 2.3. Statistical Model

The general statistical model used to determine the effect of environmental conditions on milk yield at the single animal level has the following general form:

*y_i,j_* = *DIM_i,j_* + *THI_i,j_* + *THI_i,j-1_* + *THI_i,j-2_* + *THI_i,j-3_* + *THI_i,j-4_* + *THI_i,j-5_* + *e_i,j_* (2)

where *y_i,j_* is a test-day milk yield for cow *i* at day *j*; *DIM_i,j_* denotes the effect on milk yield of the DIM of cow *i* at day *j*; *THI _i,j_* is the effect on milk yield for cow *i* of the daily average THI at day *j*; *THI_i,j_*_−*1*_*—THI_i,j_*_−*5*_, respectively, represent the effect on milk yield for cow *i* of the daily average THI at day from *j*−1 to *j*−5; *e_ij_* represents the random residual effect, a priori assumed to be independently and identically distributed as *N*(0,s_e_^2^), where s_e_^2^ is the residual variance. In particular, several statistical models have been tested also considering a longer period, starting from 10 days prior to testing. Then, it was gradually reduced to 5, removing one day at a time with the value of the average relative error that remained almost unchanged (modifications lower than about 0.1%). Only with the removal of the THI value of the fifth day prior to testing did the average error increase significantly, thus leading to the decision to consider a preceding period of 5 days. In order to predict the heat stress effects at the level of a single cow, seven different features (i.e., predictors) have been used as input data to the Random Forest algorithm, better detailed in the following section, and the dataset of each animal has been divided into data for the training phase and data for the testing phase.

### 2.4. Model Development

**Figure 3:**
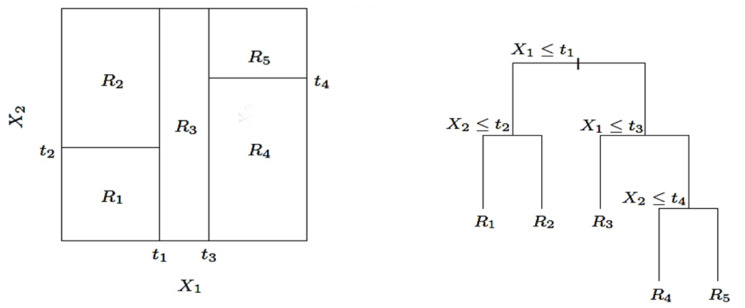
Partitions and CART. Left scheme shows a partition of a two-dimensional feature space by recursive binary splitting, as used in CART, applied to some fake data. $X_{1}$ and $X_{2}$ are sample features, $R_{i}$ is the *i-th* region of the features’ space. Right panel shows the tree corresponding to the partition. The variable *t* is a generic parameter.

The regression analysis of the collected data was performed by using the Random Forest algorithm [22], an ensemble learning method that makes predictions by averaging over the predictions provided by several independent random models [23]. The algorithm (see Figure 3) was originally conceived as a method of combining several classification and regression trees (CARTs) [24] using bagging [25], and as the name suggests, it is a tree-based ensemble with each tree depending on a collection of random variables. Random decision trees have found widespread applications thanks to several features, such as the ability to capture interactions between predictors, to deal well with irrelevant predictors, being robust in terms of outliers in the predictors and well scalable for large sample sizes [26]. In the present work, the algorithm was adopted for regression purposes by using the Scikit-Learn Python library [27] in order to establish the random forest model (RFM) best fitting the data values of each cow. A key advantage of the recursive binary tree is its interpretability. The feature space partition is fully described by a single tree. With more than two inputs, partitions such as that in the top scheme of Figure 3 are difficult to draw, but the binary tree representation works in the same way [26]. Furthermore, the Random Forest algorithm can provide useful indications on the most important predictors between those included in the training dataset. On the other hand, Random Forests are frequently used as “black box” models, as they generate reasonable predictions across a wide range of data even if they sacrifice the intrinsic interpretability present in the decision trees.

The Random Forest method is based, as for most of the data-driven methods, on the minimization of a function. Then, for a random vector *X* containing the values of the independent variable (i.e., the regressor or predictor) and a random vector *Y* collecting the values of the dependent variable (i.e., response), it is possible to assume an unknown joint distribution *P_XY_*(*X,Y*). The goal is to find a predictor function *f*(*X*) for predicting *Y*. The prediction function is determined by a loss function *L*(*Y, f*(*X*)) to be minimized. Intuitively, *L*(*Y, f*(*X*)) measures the distance between vectors *f*(*X*) and *Y*, and it should penalize values of *f*(*X*) distant from *Y*. For regression purposes, a typical choice of *L* is the squared error loss function:

*L*(*Y, f*(*X*)) = (*Y − f*(*X*))^2^ (3)

while *L* is usually a binary function (is a zero-one function in this work) for classification applications:

(4) $$L\left(Y,\;f\left(X\right)\right)=\{\begin{array}{c} 0\;if\;Y=f\left(X\right)\\ 1\;otherwise \end{array}$$

From the minimization of the loss function, the collection of the *n* base learners *b* = [*h_1_*(*X*), …, *h_n_*(*X*)] are identified. Then, they can be combined to provide the so-called ensemble predictor *f* (*X*):

(5) $$f\left(X\right)=\frac{1}{n}\overset{n}{\underset{j=1}{\sum }}h_{j}\left(X\right)$$

providing the best approximation of *Y* [28].

A significant advantage of the RFMs is the possibility of assigning a score to each individual feature composing the input of the statistical model. The scores are representative of the importance of the different features in the model output (i.e., the prediction). A function of the *scikit-learn* library allows users to produce the ranking of the features and the evaluation of various scores.

Two of the most important parameters for the application of RFMs are the size of each tree (i.e., number of nodes) and the number of trees adopted. If the parameters are too large, overfitting problems could appear, while if the values are too small for the complexity of the data, the model is not able to converge to a suitable solution. In this work, for the first parameter, a self-expanded criterion, it was assumed that the nodes number expand by itself when the number of samples is bigger than 2. Instead, the number of trees has been set equal to 1000.

The dataset of each selected cow was divided in two portions: one used for the training phase and the other for the validation, and a specific RFM was obtained for each animal. More details about the training/test division are provided in the following subsections. The RFM has been developed for the assessment of the daily yield (the dependent variable) starting from the values of the independent variables.

### 2.5. Scenarios and Performance Indicators

#### 2.5.1. Scenarios Investigated

The RFMs discussed above have been developed with the main goal to assess the daily yield of each cow. The models can be applied as a regression tool or as predictive tool. In this paper, we will show that the methodology proposed here can be applied for both purposes, and this aspect will be investigated by setting three different scenarios, i.e., A, B and C, to train and test the RFMs.

##### Scenario A

**Figure 4:**
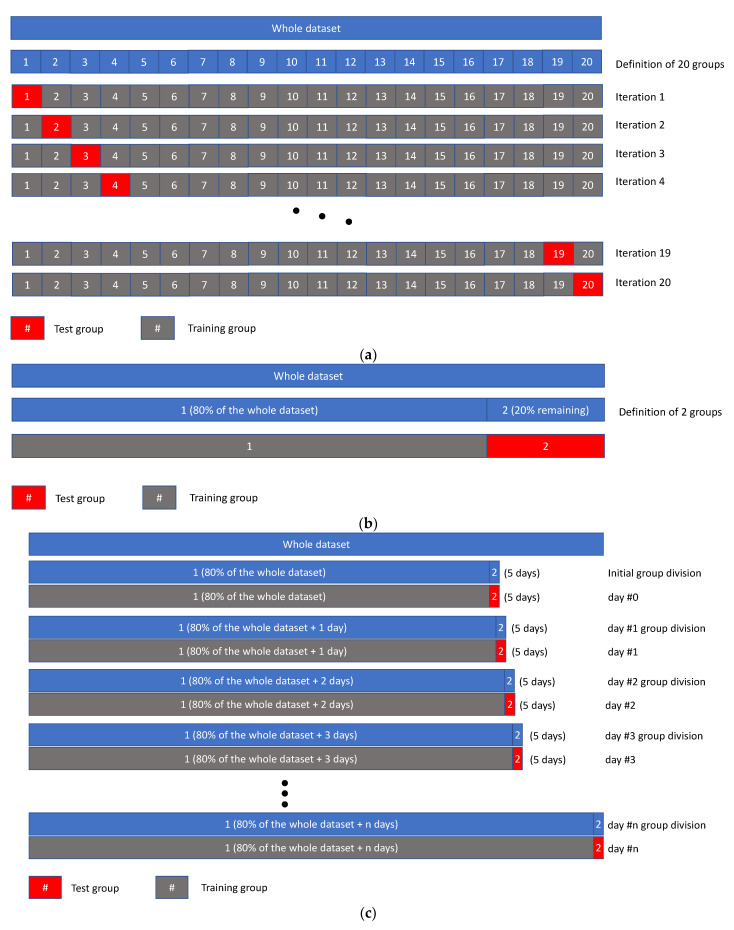
Division of the datasets for the different scenarios. (**a**) Scheme of the 20-fold cross-validation procedure used in scenario A. (**b**) Scheme of the scenario B. (**c**) Scheme of the scenario C.

Scenario A has been used to train and test the RFMs for regression purposes. In this scenario, the dataset of each cow was sampled with a cross-validation procedure, a resampling procedure evaluating machine learning models on a limited data sample. In particular, the *k*-fold cross-validation procedure [29] was considered by adopting a *k* value equal to 20 (so adopting a 20-fold cross-validation procedure). In this procedure, the dataset was divided into 20 equal parts (i.e., groups) and the training/testing process ran 20 times each time with a group used as test, the holdout group, and the others 19 groups used to train the model. In this scenario, the train and test values are randomly selected by the extraction algorithm. The accuracy of each prediction was used to evaluate the performance of the model. The scheme of the 20-fold cross-validation procedure is shown in Figure 4a.

##### Scenario B

Scenario B has been adopted with the objective to train and test an RFM for the assessment of continuous time series values by considering the need to apply the model for the assessment of future productive trends of cows under different climatic conditions. In this scenario, the dataset of each cow was divided into two groups: the initial 80% of the data were used for training while the last 20% were used to test the model accuracy and reliability (see Figure 4b). In this case, for each cow, a continuous series of daily milk yields was obtained from the model and compared to the real one.

##### Scenario C

Scenario C was obtained, starting from the scenario B, under the hypothesis that during the time, new available data are added in the training phase to improve the predictive capability of the model. This scenario would simulate the application of a RFM for the prediction of future events in a short period, i.e., 5 days, with time series also taking into account the increase in knowledge of the model that the new available data can provide. Starting from the condition of scenario B, in scenario C the RFM model is trained continuously by introducing one more day and it is adopted for the prediction of the milk yields of 5 days forward (see Figure 4c).

## 3. Results and Discussion

As discussed in the previous section, the statistical model assumed in the paper considers the daily average THI as a representative parameter of the barn thermo-hygrometric conditions. The daily milk yield of a single cow was assessed by RFM including the climatic effects of the actual day (i.e., the “day 0”) and those of the past five days (i.e., days −1, −2, −3, −4 and −5). In this way, the model can also consider the heat load duration and the cumulative effects of consecutive days on inducing animal heat stress.

**Figure 5:**
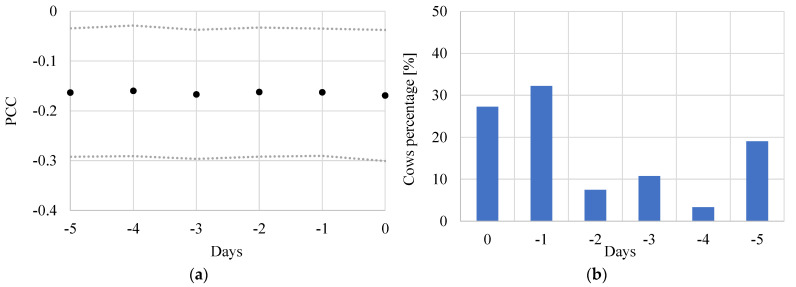
Correlation between milk yield and climatic data. (**a**) Pearson correlation coefficient: average values and average ± St. Dev. values. (**b**) Percentage distribution of cows vs. day with highest negative effects on milk production (i.e., the day with lower PCC for the single animal).

A preliminary correlation analysis has been performed with the aim to evaluate the delay between daily yield and climatic conditions. For the 91 cows considered in the study, the Pearson correlation coefficient (PCC) has been established between the milk yield and THI_0_, THI_−1_, THI_−2_, THI_−3_, THI_−4_ and THI_−5_. The PCC values are reported in Figure 5a for the various days. The values reported are the average on the 91 animals and the average ± standard deviation (St. Dev.). The trends showed that a weak negative correlation, similar for the different days, exists but is not possible to establish the day with the highest correlation as the different days have similar PCC values. This is because, in the herd, two different cow groups exist. In fact, about 60% of the cows were more sensitive to THI_0_ and THI_−1_, while the other 40% have daily yields more affected by the THI_−2_ to THI_−5_ and, for this group, it is evident that heat stress causes effects with a delay of 3–5 days. Figure 5b shows the animal percentage vs. day with highest yield decrease. Summarizing, in order to be able to catch the daily milk yield of every single cow, the climatic data up to 5 days before the day of interest have been introduced in the numerical model as independent variables.

### 3.1. Goodness-of-Fit (Scenario A)

#### 3.1.1. Cow Level

**Figure 6:**
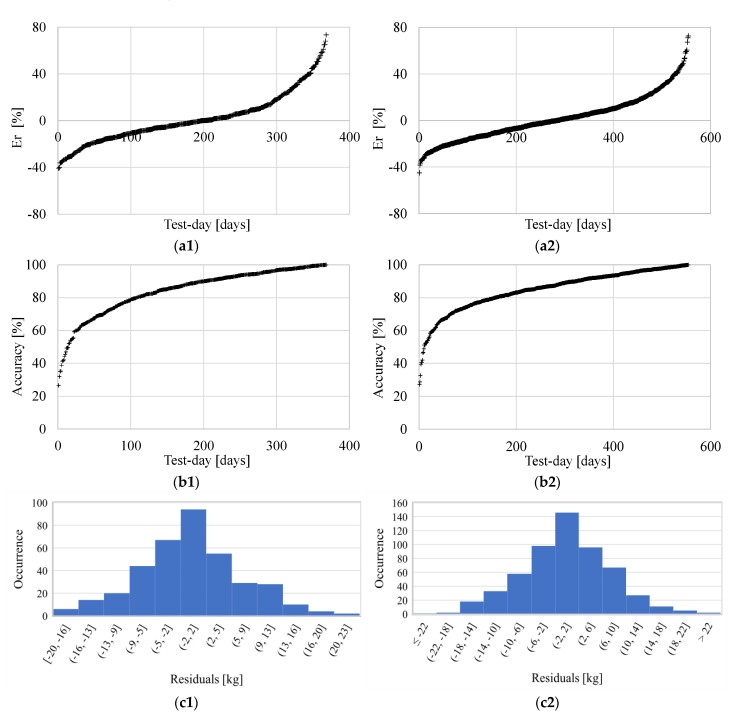
Main results obtained for scenario A for two representative cows where (**1**) represents animal #226 and (**2**) animal #243. (**a**) Relative error Er of the daily yield in ascending order; (**b**) accuracies of the daily yield predictions in ascending order; (**c**) histogram representation of the residuals of the predictions.

Firstly, the responses of the RFMs, applied in Scenario A for regression purposes, are reported at the single cow level. In this regard, and for the sake of brevity, an extended description of the results has been reported only for two cows, randomly selected in the herd in order to provide the general validity of the results. The two animals are #226 and #243 (the codes adopted by the farmer have been maintained). They have 360 and 543 test-days, respectively. To establish the goodness-of-fit of the models, the trends of the relative error Er on the daily milk yield are shown in ascending order in Figure 6a, while the prediction accuracies are showed in Figure 6b. The minimum and the maximum Er are about −40% to −70% respectively, but most of the daily predictions are characterized by a high accuracy. In fact, for animal #226, about 58% and 28% (i.e., 210 and 100 out of 360 values) of the predictions provided good and very good accuracies, respectively. Similarly, for animal #243, about 57% and 27% (i.e., 307 and 148 out of 543 values) of the predictions had good and very good accuracies, respectively. For the two cows, the average accuracies (median ± standard deviation) are 88.64% ± 14.31% and 87.20% ± 12.58%. Moreover, from Figure 6c, it is evident that the residuals have normal distribution centered on the zero value. In fact, Er for the sum of the daily yields for the test days is very low, i.e., +0.29% and −0.64%, for the two animals described here. This is an important aspect, as it confirms that the trained RFMs can assess, for each single animal, the daily milk production with a good accuracy and with a predicted yield trend, on average, close to the real yield trend.

#### 3.1.2. Analysis of Variability within the Herd

**Figure 7:**
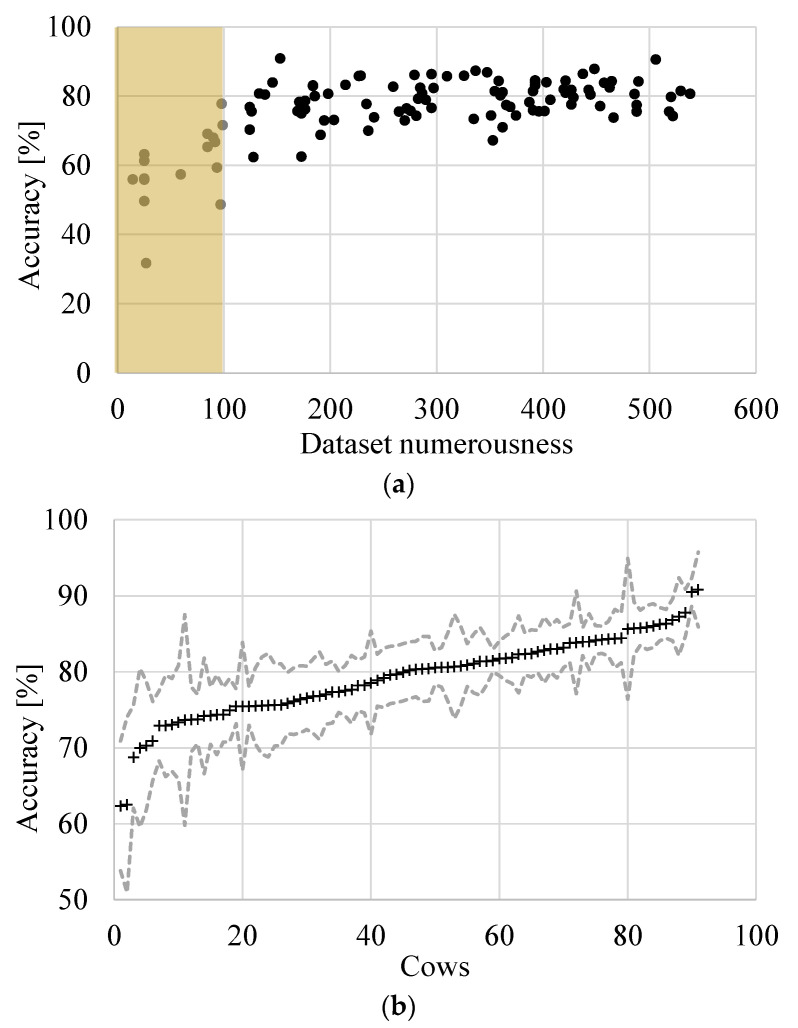
Main results obtained for scenario A for the 91 cows of the study. (**a**) Median accuracy for each cow vs. dataset numerousness; (**b**) median accuracy ± standard deviation for each cow sorted ascendingly.

The comparison of the results for a single cow (cow level) can be used to define the variability within the herd, so considering the cow-to-cow variability. In this regard, the median accuracies of the daily yields, of each cow, are showed in Figure 7a vs. the data numerousness (i.e., the test-day number of each cow, different from cow to cow).

The figure highlights that for the 91 cows considered in the study, having data numerousness higher than 100 days, the median accuracy for the different animals ranges between 63% and 92%. For the sake of completeness, the figure also depicts the median accuracy of the cows not considered in the work (i.e., cows with less than 100-day dataset numerousness). It is rather clear that a reduced number of events could represent a problematic aspect for the training phase of the RFM and, for this, only 91 animal datasets have been considered robust for the purpose of the work.

As far as the cows’ datasets bigger than 100 days are concerned, the accuracy values do not increase with the numerousness of dataset, and this leads to the belief that even by enlarging the yield dataset, the average accuracy is not likely to increase significantly. This uncertainty is probably difficult to remove since it can be attributed to the variability in the cow’s response, which is not only governed by environmental conditions, but other animal welfare factors could contribute.

Then, in Figure 7b, the median accuracies ± standard deviation of the 91 cows with datasets bigger than 100 test days are reported in ascending order. The average (out of the cows) median accuracy of the predictions is equal to 79.26%, whereas the standard deviation of the median accuracy is 5.33%.

**Figure 8:**
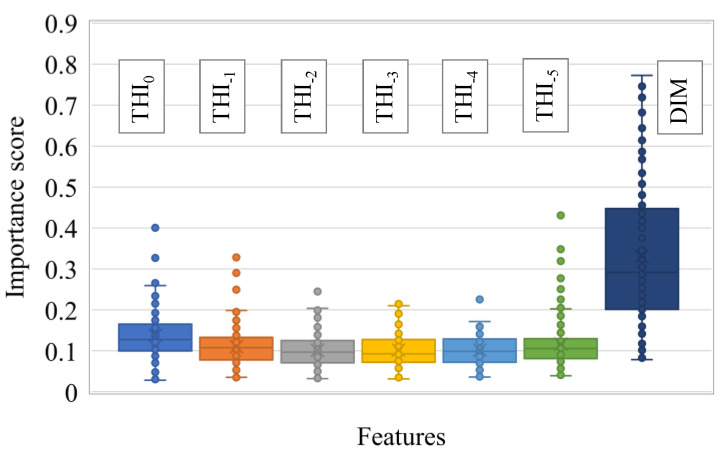
Boxplot diagram of the importance score of the different features for the whole dataset in scenario A.

Finally, the analysis of the importance score (IS) of the different features is reported in the following. The IS of the variables represent the key aspect of the RFM since, by means of the IS it is possible to hierarchize the features of the statistical model by attributing different scores to the various independent variables. In Figure 8, the boxplot diagram of the different ISs is reported for the dataset containing the 91 investigated cows.

Moreover, the most important values of the diagram are summarized in Table 1, collecting, for each independent variable (feature), the minimum value, the maximum value, the median value, the standard deviation value, and the coefficient of variation (CoV) value obtained for the IS. In Table 1, as expected, it appears to be clear as the DIM has the highest score, with a median IS = 0.29. Then, THI_0_, i.e., the average THI of the day to predict (median IS = 0.13), whereas the other features (THI_−1_–THI_−5_) have comparable median ISs, ranging from 0.090 to 0.099. The minimum and maximum values recorded for the different features circumscribe large ranges, confirming the high cow-to-cow variability of the RFMs. The features with the highest median IS values are, at the same time, those associated with highest CoV values.

**Figure 9:**
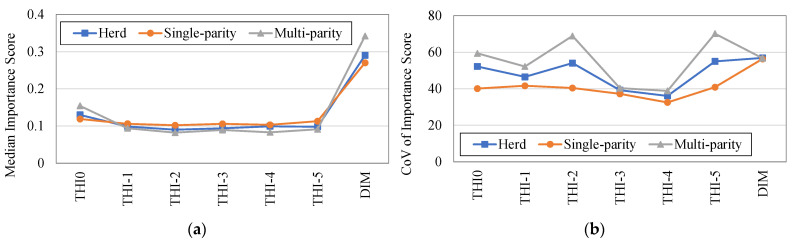
Disaggregation of the IS values between single-parity and multiparity cows. (**a**) Median value of each IS; (**b**) CoV value of each IS.

Lastly, Figure 9 reports the trends of median and CoV values of each IS disaggregated into single-parity and multiparity cows. From these preliminary results, it seems that the ISs are not dependent, in terms of median values, on the parity number and in general they are quite homogeneous and representative of the whole herd. On the other hand, the presence of cows with different parity numbers may increase the cow-to-cow variability of the IS values of the multiparity group.

### 3.2. Milk Yield Predictions (Scenario B and Scenario C)

#### 3.2.1. Scenario B

**Figure 10:**
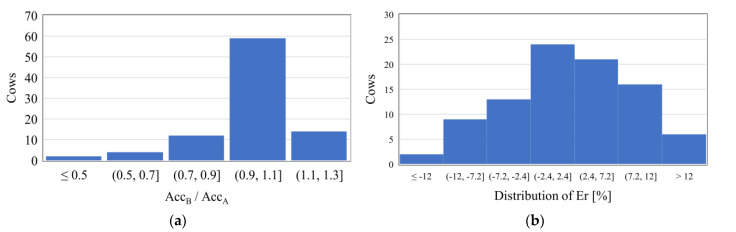
Performance indicators for scenario B. (**a**) Histogram distribution of the ratio between the average accuracy obtained in scenarios B and A for the same cow; (**b**) distribution of the error Er on the sum of the daily yields over the test days.

In this scenario, the Random Forest model was used to assess future milk yields. The same database as for scenario A has been used, even if with different division between training and testing. As far as the average accuracy related to single cow is concerned, it has very similar results to those obtained for the same animal in scenario A. For the sake of a general comparison, the histogram distribution of the ratio Acc_B_/Acc_A_, i.e., the ratio between the average accuracy obtained in scenarios B and A, for the same cow, is depicted in Figure 10a. For 59 cows out of 91, i.e., 65% of the analyzed animals, the ratio ranges from 0.9 to 1.1, and for 93% of the cows it ranges from 0.7 to 1.3. Thus, the accuracies of the predictions of scenario A and scenario B appear to be very similar and the RFMs provide comparable precision levels. In scenario B, the average (out of the cows) median accuracy of the predictions is equal to 81.91% (equivalent to Er = 18%), whereas the standard deviation of the median accuracy is 13.02%. confirming a generally good accuracy, even if it is slightly more scattered than scenario A.

As a confirmation of the good accuracy of the models, Figure 10b displays the distribution of Er on the sum of the daily yields over the period of tests. For 80% of the animals, the Er value is included in the range ±10%, with an average value of the cows equal to 1.85%. This means that, if we sum the daily yield of each cow for the test days (68 days on average), the relative error in the assessment of the total milk production is lower than 2%.

**Figure 11:**
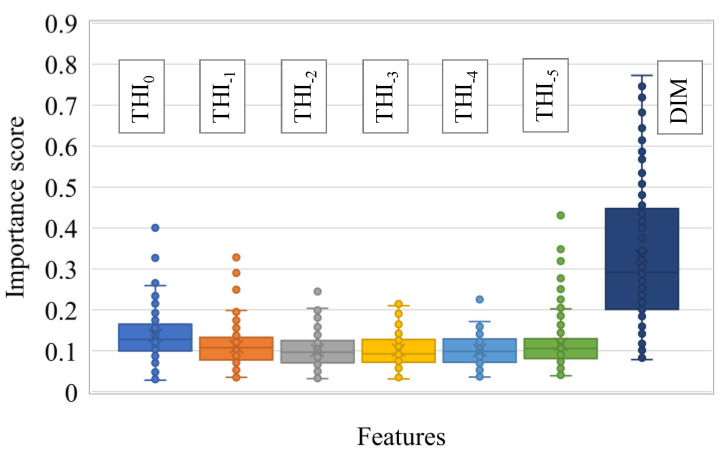
Boxplot diagram of the importance score of the different features for the whole dataset in scenario B.

Lastly, the boxplot diagram of the different ISs is reported in Figure 11 for the whole dataset containing the 91 investigated cows for scenario B and the most representative values of the diagram are collected in Table 2.

The DIM has the highest importance scores, with a median IS = 0.29. Then, THI_0_, i.e., the average THI of the day to predict, has a median of IS = 0.13, whereas the other features (THI_−1_–THI_−5_) have comparable median ISs ranging from 0.093 to 0.11. Moreover, the feature with highest median IS value (DIM) is affected by the highest variability in the IS values (i.e., highest values of CoV).

#### 3.2.2. Scenario C

In scenario C, the new available data can improve the model predictive capability since new data are introduced in the training phase. In this scenario, milk yield predictions for a short-term period of 5 days have been evaluated with the measured values for each cow. Then, the average relative error on the five daily yields (Er_5_) has been evaluated for each subcase obtained by pushing forward the training phase 1 day at a time, as represented in Figure 4c. The added value of this scenario is that it can monitor the evolution, over the time, of the performance indicators by giving further purposes to the RFMs developed here. In this way, it is possible to establish the effects of the new data introduced in the training dataset and evaluate the trend of Er_5_ due to the training window increase.

**Figure 12:**
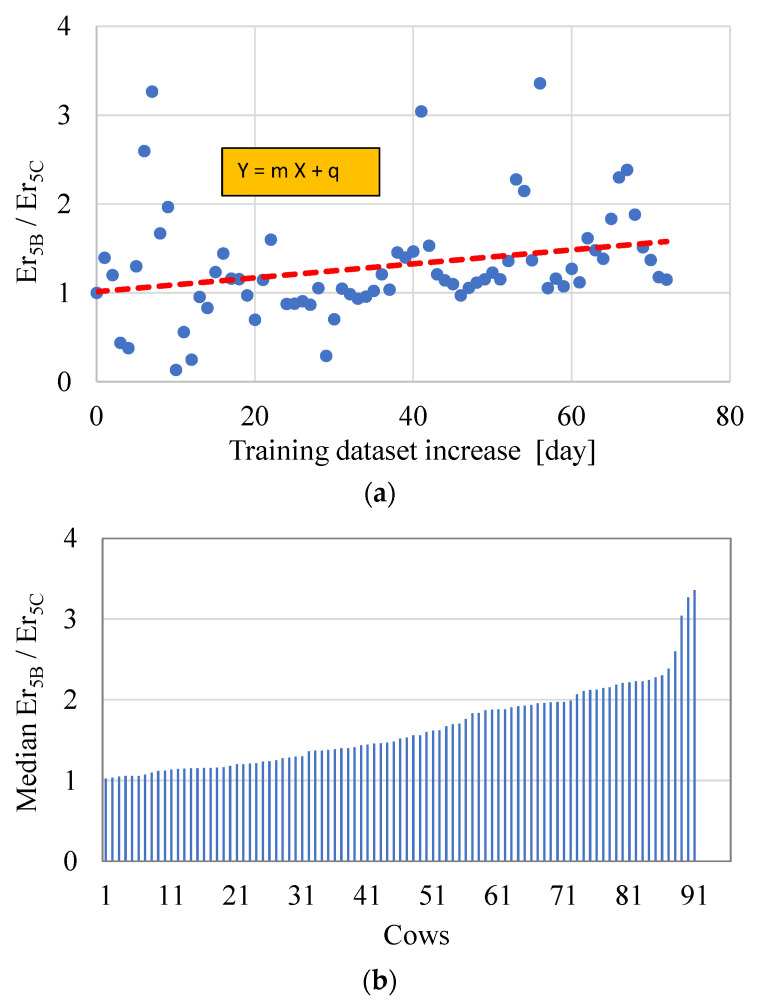
Analysis of the results on the ratio Er_5B_/Er_5C_. (**a**) Evolution of the ratio for the cow #248 with training dataset increase. (**b**) Median ratios in ascending order for the 91 cows.

As a representative example, Figure 12a displays, for a generic cow (#248), the evolution of the values of the ratio Er_5B_/Er_5C_, between the values of Er_5_ calculated in an analogous way for the scenarios B and C, respectively. As the figure shows, the values are rather scattered, but the general tendency of the trend is to increase with respect to the value recorded at the beginning of the series (i.e., for a value of the training dataset increase equal to 0, the ratio must be equal to 1). The positive effect during the time can be evaluated as a whole and qualitatively by the slope (m) of the best fitting linear equation. If the value of m is considerably higher/lower than 0, it means that the model is improving/worsening its accuracy. Instead, values of m around 0 indicate a substantial stability of the accuracy of the predictions. This parameter, even if very intuitive, does not have a physical explanation and cannot be related in a simple way to an increase in accuracy. Therefore, for practical reasons, the increase in the predictive capability of the models has been numerically evaluated in terms of median ratio Er_5B_/Er_5C_ for each cow (see Figure 12b). Globally, the median ratio for the different cows goes from 1.02 to 3.35, with a mean value for the 91 cows equal to 1.64. Then, the augment in knowledge of the models, as expected, can increase, in a significant way, the accuracy of the predictions.

**Figure 13:**
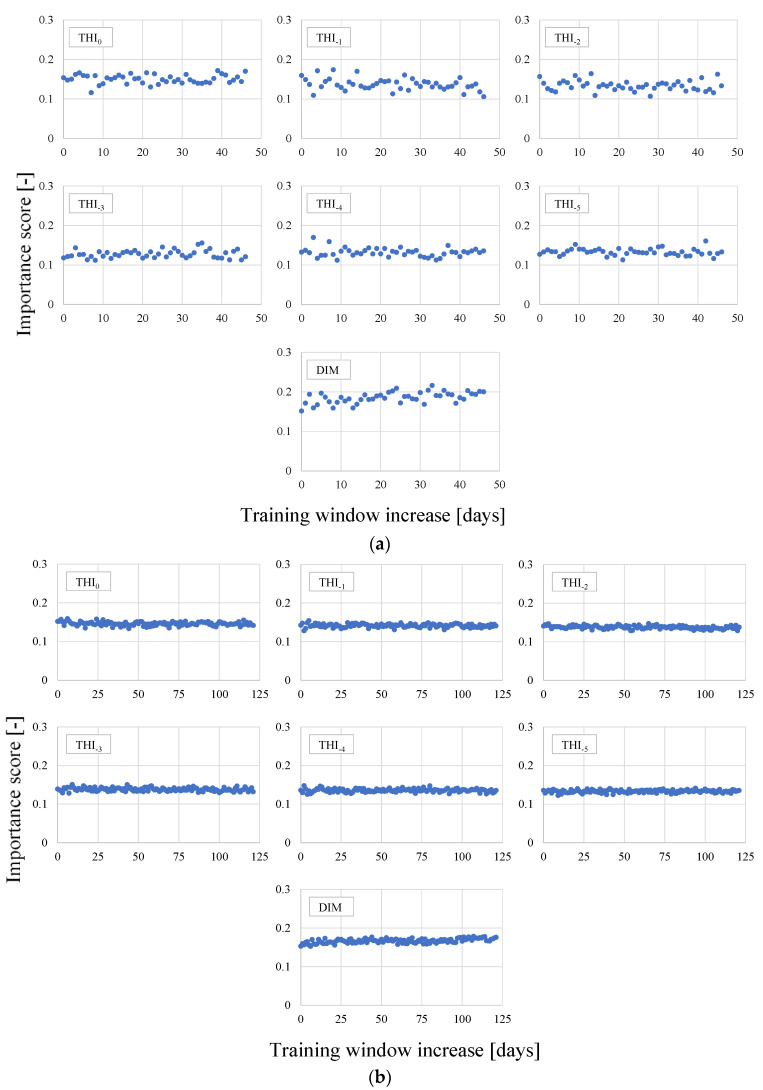
Trends of each importance score vs. training window increase for cows (**a**) #26 and (**b**) #85.

A further interesting aspect comes from the analysis of the trends of the ISs along the time. Figure 13 displays the trends obtained from each IS for two animals, i.e., #26 and #85, having different dataset sizes.

Cow #26 has milk yield data covering 180 days while cow #85 has data from 484 days. For both cows, the IS values are rather stable in the monitored period. Similar considerations can be drawn for the other investigated animals even if their results are not reported here for brevity.

## 4. Conclusions

The study aimed to define and test a Random Forest-based model for the assessment of the daily milk yield at the single cow level. The model has been applied to the data collected in two years, 2016 and 2017, in a dairy farm, located in northern Italy, and collected both productive data from the automatic milking system and environmental data from two thermo-hygrometric sensors. The statistical model used for the interpretation of the collected data is composed of seven predictors: days in milk of the cow, daily average THI of the day of the assessment and those of past five days.

The results in the paper showed that the model can detect the drop in the cow’s milk yield due to extreme hot conditions inducing heat stress effects. In fact, the average relative error provided by the model in the predictions, is about 18% with a single daily yield, whereas it becomes just 2% if the total milk production in the test days is considered. The outcomes reported in the study seem to be particularly relevant for three main reasons:

1. the size of the training dataset adopted in the analysis is suitable for the objective of the study;
2. the statistical model assumed in the study seems suitable for the work;
3. the RFM developed by the regression procedure is rather robust and reliable with respect to the type of data.

Then, the results confirm that the obtained RFM can represent a reliable and viable tool for the evaluation of future productive scenarios of dairy cows in the presence of heat stress effects. This could help to develop and improve decision support for farmers to increase both milk yield and animal welfare and, on the other hand, to reduce the resources needed, so to increase the sustainability of the dairy sector.

## Figures and Tables

**Table 1:** Minimum value, maximum value, median value, standard deviation value, and coefficient of variation (CoV) value of the ISs obtained for the different independent variables calculated for the whole dataset in scenario A.

	THI_0_	THI_−1_	THI_−2_	THI_−3_	THI_−4_	THI_−5_	DIM
Min	0.023	0.024	0.024	0.028	0.020	0.025	0.086
Max	0.561	0.287	0.435	0.204	0.175	0.444	0.821
Median	0.130	0.099	0.090	0.094	0.099	0.098	0.290
St. Dev.	0.075	0.049	0.056	0.039	0.034	0.061	0.197
CoV [%]	57.692	46.483	54.057	39.206	36.103	55.003	56.850

**Table 2:** Minimum value, maximum value, median value, standard deviation value, and coefficient of variation (CoV) value of the ISs obtained for the different independent variables calculated for the whole dataset in scenario B.

	THI_0_	THI_−1_	THI_−2_	THI_−3_	THI_−4_	THI_−5_	DIM
Min	0.028	0.035	0.032	0.032	0.036	0.039	0.079
Max	0.416	0.328	0.245	0.230	0.238	0.431	0.772
Median	0.128	0.108	0.097	0.093	0.099	0.106	0.291
St. Dev.	0.065	0.045	0.039	0.041	0.039	0.057	0.172
CoV [%]	50.915	41.595	40.595	43.999	39.314	54.174	58.964

## Data Availability

Restrictions apply to the availability of these data. Data was obtained from the farm *Azienda Agricola Piazzi* and are available from the authors with the permission of *Azienda Agricola Piazzi*.
